# Carbon Monoxide as a Signaling Molecule in Plants

**DOI:** 10.3389/fpls.2016.00572

**Published:** 2016-04-29

**Authors:** Meng Wang, Weibiao Liao

**Affiliations:** College of Horticulture, Gansu Agricultural University, LanzhouChina

**Keywords:** abiotic stress, carbon monoxide (CO), growth and development, antioxidant defense, physiological role, signaling transduction

## Abstract

Carbon monoxide (CO), a gaseous molecule, has emerged as a signaling molecule in plants, due to its ability to trigger a series of physiological reactions. This article provides a brief update on the synthesis of CO, its physiological functions in plant growth and development, as well as its roles in abiotic stress tolerance such as drought, salt, ultraviolet radiation, and heavy metal stress. CO has positive effects on seed germination, root development, and stomatal closure. Also, CO can enhance plant abiotic stress resistance commonly through the enhancement of antioxidant defense system. Moreover, CO shows cross talk with other signaling molecules including NO, phytohormones (IAA, ABA, and GA) and other gas signaling molecules (H_2_S, H_2_, CH_4_).

## Introduction

Carbon monoxide (CO), which has long been widely considered as a poisonous gas (“the silent killer”) since 17th century, is a low molecular weight diatomic gas that occurs ubiquitously in nature. However, CO has been recently proven to be one of the most essential cellular components regulating a variety of biological processes both in animals and plants (Xie et al., 2008). Generally speaking, CO arises in biological systems principally during heme degradation as the oxidation product of the α-methene bridge of heme, and this process is catalyzed by heme oxygenase enzymes (HOs, EC 1.14.14.18; Bilban et al., 2008). CO plays a critical role as neurotransmitter (Boehning et al., 2003), inhibitor of platelet aggregation (Brüne and Ullrich, 1987) and suppressor of acute hypertensive (Motterlini et al., 1998) in animals. Similarly, involvement of CO gas in different biological processes has also been found in plants. For instance, it acts as a compound with hormonal effects, affecting seed germination (Dekker and Hargrove, 2002), root development (Cui et al., 2015), and inducing stomatal closure (Cao et al., 2007a). In natural environments, plants develop inducible defence systems to survive biotic and abiotic threats, thus producing a wide variety of defense-related hormones to unlock the defense-related regulatory networks. CO is also generated against oxidant damage under abiotic stress, such as drought stress (Liu et al., 2010), salt stress (Ling et al., 2009), and heavy metal stress (Meng et al., 2011). In addition, CO not only acts as a signaling molecule during plant growth and development, but also interacts with other signaling molecules in plant stress response, growth and development (Santa-Cruz et al., 2010; Lin et al., 2014; Xie et al., 2014). In view of the evidence described above, we provide a brief update here on CO synthesis, physiological function in plant growth and development and its response to abiotic stresses. Furthermore, the cross-talk between CO and other signaling molecules including phytohormone, hydrogen peroxide (H_2_O_2_), and other small gas signaling molecules is also discussed.

## Synthesis of CO in Plants

**FIGURE 1:**
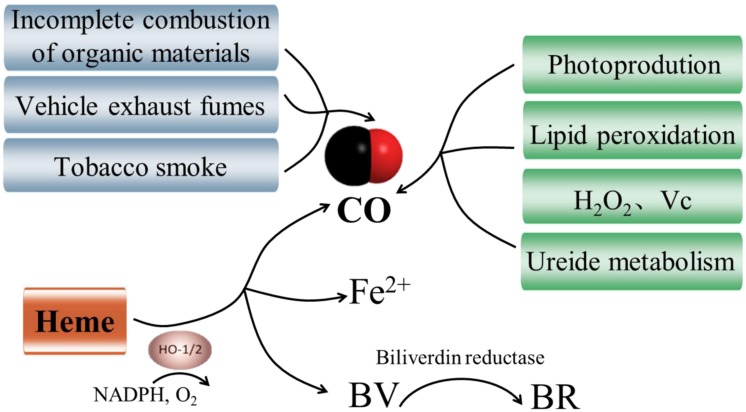
**Pathways of generation of carbon monoxide (CO).** There are three synthetic routes for CO. Heme source is viewed as the main productive route of CO in animals and plants (Bilban et al., 2008). CO is also generated via incomplete combustion of organic materials, tobacco smoke and vehicle exhaust fumes in atmosphere. Additionally, non-heme sources including photoproduction (Schade et al., 1999), lipid peroxidation (Zilli et al., 2014), hydrogen peroxide (H_2_O_2_) and ascorbic acid (Vc; Dulak and Józkowicz, 2003), and ureide metabolism have been proposed in plants (Zilli et al., 2014).

Carbon monoxide is primarily generated by incomplete combustion of organic materials in atmosphere, and it is also a significant component of tobacco smoke and vehicle exhaust fumes (**Figure 1**). In animals and plants, the generation of intracellular CO and its actions are closely connected with HOs. As shown in **Figure 1**, HOs catalyze the oxidative conversion of heme to CO, free iron (Fe^2+^), and biliverdin (BV) in presence of molecular oxygen and electrons supplied by NADPH (Bilban et al., 2008). BV is then converted to the potent antioxidant bilirubin (BR) by biliverdin reductase. To date, three isoforms of HO have been detected in animals, including HO1 (32 kDa), HO2 (36 kDa), and HO3 (33 kDa) (Maines, 1997). HO1 is the highly inducible isozyme which increases rapidly to diverse stimuli and protects tissues against a wide range of injuries (Ryter and Choi, 2015). HO2 and HO3 are constitutively expressed with very low activity. Although the most investigated mechanism for CO production in animals involves HOs, much smaller amounts of CO can derive from other sources, like lipid peroxidation (Vreman et al., 2001; **Figure 1**).

In plants, the presence of CO biosynthesis was first reported by Wilks (1959) and subsequently the photoproduction of CO in living plants was also identified (Schade et al., 1999; **Figure 1**). Furthermore, Muramoto et al. (2002) found a plastid heme oxygenase (AtHO1) recombinant protein which was able to catalyze the formation of CO from heme molecules *in vitro*. To date, HOs are still viewed as the main enzymatic source of CO in plants (Xuan et al., 2008; **Figure 1**). Recent researches provided exciting evidence that the genes for HOs have been identified in a variety of plant species (Emborg et al., 2006; Wang et al., 2014). It comprises a small family with four members in total, which can be classified into two sub-families: HY1 (HO1), HO3 and HO4 all belong to the HO1 sub-family, while HO2 is the only number of the HO2 sub-family (Shekhawat and Verma, 2010). All members of the HO1 sub-family (HY1, HO3, and HO4) can convert heme to BV with a concomitant release of CO and Fe^2+^, whereas HO2 sub-family does not exhibit HO activity (**Figure 1**). Despite the enzymatically catalyzed reaction by HOs has been considered as the main productive route of CO in plants, the opposite results have been proposed in soybean (Zilli et al., 2014). The authors suggested that HOs are not the main source of CO in soybean plants, and lipid peroxidation and ureide metabolism could also be considered as potential sources of CO (**Figure 1**). Additionally, heme methylene bridges could be broken and CO released when exogenous H_2_O_2_ or ascorbic acid was supplied (Dulak and Józkowicz, 2003; **Figure 1**). Collectively, CO synthetic is a complex physiological process and more non-enzymatic biosynthetic processes of CO need to be elucidated.

## Role of CO in Plant Growth and Development

A high level of exogenous CO is toxic in plants and animals, however, CO at a proper level involves in many important physiological processes as an active signaling mediator. In animals, the amazing progress in our understanding of the biology of CO has developed rapidly. It was convincingly reported that exogenous CO gas could exert the beneficial effects on modulating a number of physiological events including neurotransmission (Boehning et al., 2003), vasodilation (Motterlini, 2007), and platelet aggregation (Brüne and Ullrich, 1987). More importantly, application of exogenous CO is developing a new therapeutic strategy for treatment of numerous clinical conditions (Fagone et al., 2015). Also in plants, CO has been studied to elucidate the roles of this enigmatic signaling molecule in plant growth and development. Accumulating evidence in plants has shown that CO is used for a number of intercellular and intracellular biological functions. For instance, CO was likely to delay gibberellins (GA)-triggered programmed cell death (PCD) in wheat aleurone cells by up-regulating of *ascorbate peroxidase (APX) and catalase (CAT)* expression, and decreasing H_2_O_2_ overproduction (Wu et al., 2010; **Table 1**). Until now, the studies of roles of CO in plants mostly focus on seed germination, root development, and stomatal closure.

**Table 1:** Overview of CO-mediated physiological processes in plants.

Physiological process	Plant species	Tissue	CO-induced effect	Reference
Seed germination	*Setaria faberii*	Seed	+	Dekker and Hargrove, 2002
	*Oryza sativa*			Liu et al., 2007
	*Triticum aestivum*			Liu et al., 2010
	*Brassica nigra*			Amooaghaie et al., 2015
Lateral root formation	*Solanum lycopersicum* *Brassica napus*	LR	+	Guo et al., 2008 Cao et al., 2007b
Adventitious root development	*Cucumis sativus* *Phaseolus radiates* *Cucumis sativus* *Cucumis sativus* *Cucumis sativus*	AR	+	Xuan et al., 2008 Xu J. et al., 2006 Cui et al., 2015 Lin et al., 2014 Xuan et al., 2012
Root hair development	*Solanum lycopersicum*	Root hair	+	Guo et al., 2009
Root elongation	*Triticum aestivum*	Root tip segments	+	Xuan et al., 2007
Programmed cell death	*Triticum aestivum*	Aleurone layers	-	Wu et al., 2010
Stomatal closure	*Vicia faba*	Leaf	+	Cao et al., 2007a; She and Song, 2008; Song et al., 2008

### Seed Germination

Seed germination, which is a highly specialized phase in plant life, is essential for seedling establishment. It is a critical step in a plant’s life cycle and is regulated by a wide range of endogenous and environment factors (Kong et al., 2015). Several researches demonstrated that CO exerted an advantageous effect on promoting seed germination in a dose-dependent manner and in many plants. The application of low levels of exogenous CO (0.1 or 1%) stimulated seed germination of foxtail (*Setaria faberi*) under favorable temperature and moisture conditions, while germination decreased with the addition of 75% CO due to the inhibition of mitochondrial respiration (Dekker and Hargrove, 2002; **Table 1**). Both CO donor heme and CO aqueous dose-dependently accelerated the physiological process of seed germination in *Oryza sativa* via activating amylase activity and increasing the formation of energy resources (Liu et al., 2007; **Table 1**). Similarly, CO as a positive regulator was also involved in the process of seed germination in wheat (Liu et al., 2010) and *Brassica nigra* (Amooaghaie et al., 2015; **Table 1**).

### Root Development

The root systems have been identified to play important roles in plant nutrient and water acquisition. CO has exhibited positive effects on regulating plant root development. For example, the promoting effects of auxin (IAA) or nitric oxide (NO) on root elongation were mimicked by application of aqueous solution of CO with different saturations in wheat seedlings (Xuan et al., 2007; **Table 1**). In tomato, exogenous CO promoted root hair density and elongation, which increased 3.38- and 2.48-folds compared with the control. Genetic analyses have shown that CO was able to affect the root hair formation by up-regulating *LeExt1* gene expression (Guo et al., 2009; **Table 1**). Actually, previous studies of CO-induced root development mostly concentrated on lateral root (LR) and adventitious root (AR).

#### LR Development

Lateral root is derived from the pericycle of parent root, in which mature cells are stimulated to dedifferentiate and proliferate to form a LR primordium, finally leading to the emergence of LR. LR plays an indispensable role in the development of plant root system responsible for water-use efficiency and the extraction of nutrients from soils (Guo et al., 2008). CO has been shown to induce the formation of LR. In rapeseed seedlings, the total length and number of LR increased significantly in a dose-dependent manner with the CO donor hematin or CO aqueous, while the positive effects were fully reversed by the addition of the CO scavenger hemoglobin (Hb) or the CO-specific synthetic inhibitor zinc protoporphyrin-IX (ZnPPIX; Cao et al., 2007b; **Table 1**). Treatment with exogenous CO up-regulated heme oxygenase-1 (*LeHO-1*) expression and the amount of LeHO-1 proteins, then stimulated the formation of tomato LR (Guo et al., 2008; **Table 1**). Above results indicate that exogenous CO is, at least partially, correlated with the formation process of LR in plants.

#### AR Development

Adventitious root development is an essential step for vegetative propagation which involves the reestablishment of a meristematic tissue after removal of the primary root system (Liao et al., 2012). AR formation is affected by multiple endogenous and exogenous factors, wherein IAA is viewed as one of the most important phytohormones in mediating AR (Xuan et al., 2008). The investigation of CO-induced AR formation can be dated back to the year 2006 that CO exhibited positive effects on AR formation in mung bean seedling (Xu J. et al., 2006; **Table 1**). Then, more attention has been given to highlight AR formation induced by CO. Xuan et al. (2008) discovered that CO dose-dependently promoted AR number and length in IAA-depleted cucumber seedlings by up-regulating the expression of target genes (*CSDNAJ-1* and *CSCDPK1/5*) during AR (**Table 1**). It has also been demonstrated that the induction of AR formation by methane-rich water (MRW) was blocked by ZnPPIX, and further reversed by CO aqueous (Cui et al., 2015; **Table 1**). In addition, CO could up-regulate NO production, and thereafter promoting AR formation in IAA-depleted seedlings (Xuan et al., 2012; **Table 1**). Previous results also exposed that endogenous HO-1 might be involved in hydrogen-rich water (HRW)-induced AR formation in cucumber explants (Lin et al., 2014; **Table 1**). Thus, HRW or NO-induced AR formation may require the involvement of CO (Xuan et al., 2012; Lin et al., 2014).

### Stomatal Closure

Stomatal movement critically controls the plant water status, and it can be triggered by numerous environment or hormonal factors. Among these, the stress hormone abscisic acid (ABA) is a key player in regulating stomatal movement under drought and humidity stress (Grondin et al., 2015). ABA treatment was found to increase CO content and HO activity in *vicia faba* leaves, and then researchers began to investigate the relationship between CO and stomatal closure. Interestingly, further results showed that exogenously applied hematin and CO aqueous not only resulted in the enhancement of CO release, but also induced stomatal closure in dose- and time-dependent manners (Cao et al., 2007a; **Table 1**). The CO effects in stomatal movement are similar to NO and H_2_O_2_ (She and Song, 2008; Song et al., 2008; **Table 1**).

## Response of CO in Abiotic Stress

Abiotic stresses are major constraint to plant growth, survival, yield, and distribution, which also result in the oxidative stress and reactive oxygen species (ROS) overproduction by disrupting cellular redox homeostasis. It has been known that ABA is a key regulator involved in plant developmental processes and responses to biotic and abiotic stresses (Raghavendra et al., 2010). Similar to ABA, CO is also required for the alleviation of abiotic stress-induced oxidative stress (Cao et al., 2007a).

### Salt Stress

Salt stress has become an ever-present threat to crop yields often causing many unfortunate consequences in plants, such as growth inhibition, ionic phyto-toxicity and ROS overproduction. Low concentrations of CO alleviated the inhibition of seed germination and the damage of seedling leaves produced by salt stress through enhancing antioxidant enzyme activities including superoxide dismutase (SOD), CAT, APX, and guaiacol peroxidase (GPOX) in wheat (Huang et al., 2006; Xu S. et al., 2006; **Table 2**). Similar result was confirmed in rice. CO enhanced the activities of CAT and SOD and up-regulated the expression of *CAT* and *Cu/Zn-SOD* genes, thus resulting in alleviating salt-induced oxidative damage and finally decreasing the inhibition of seed germination (Liu et al., 2007; **Table 2**). CO might increase the tolerance of wheat seedling to salt stress, and its alleviation of PCD and root growth inhibition was linked to the maintenance of ion homeostasis and the decrease of superoxide anion (O_2_^-^) overproduction (Xie et al., 2008; Ling et al., 2009; **Table 2**). In *Cassia obtusifolia*, hematin or CO-saturated aqueous solution increased the level of cytosolic osmotic substances (total soluble sugars, free proline, and soluble protein) and antioxidant enzyme activities (SOD, POD, CAT, and APX), and lightened the damage of photosynthetic system under salt stress, consequently alleviating the inhibition of seed germination and seedling growth deriving from salinity stress (Zhang et al., 2012; **Table 2**).

**Table 2:** Overview of the responses of CO in abiotic stress.

Plant species	Tissue	Abiotic stress	CO-mediated effect	CO-mediated antioxidant enzyme	CO-mediated gene	Reference
*Triticum aestivum*	Seed	Drought	Maintain antioxidative capability/ROS-scavenging activity	CAT, APX, SOD, DHAR	*HO-1*	Liu et al., 2010
*Triticum aestivum*	Seed	Salt	Counteract lipid peroxidation	CAT, APX, SOD, GPOX	–	Xu S. et al., 2006
*Triticum aestivum*	Leaf	Salt	Alleviate oxidative damage	CAT, APX, SOD, GPOX	–	Huang et al., 2006
*Oryza sativa*	Seed	Salt	Alleviate oxidative damage	CAT, SOD	*HO-1*,*CAT*, *SOD*, *APX*	Liu et al., 2007
*Triticum aestivum*	Root	Salt	Maintain ion homeostasis/up-regulate antioxidant defense	APX, GR, SOD, MDHAR, DHAR	*SOD*,*GR*, *DHAR*	Xie et al., 2008
*Triticum aestivum*	Root	Salt	Inhibition superoxide anion overproduction	SOD	*SOD*	Ling et al., 2009
*Cassia obtusifolia*	Seeds/seedlings	Salt	Increase osmotic substances/antioxidant enzyme activities	SOD, POD, CAT, APX	–	Zhang et al., 2012
*Glycine max*	Leaf	UVB	Prevent oxidative stress	CAT, APX	*HO-1*	Yannarelli et al., 2006
*Medicago sativa*	Root	Hg	Alleviate oxidative damage	GR, MDHAR, SOD	*HO-1/2*	Han et al., 2007
*Chlamydomonas reinhardtii*	–	Hg	Suppress reactive oxygen species	SOD, CAT, APX	*HO-1*	Wei et al., 2011
*Brassica juncea*	Root	Hg	Alleviate oxidative stress	SOD, POD, CAT, APX	*SOD, POD, CAT, APX*	Meng et al., 2011
*Medicago sativa*	Root	Cd	Alleviate oxidative damage	SOD, POD, APX, GR	*HO-1*, *APX, GR*	Han et al., 2008
*Chlamydomonas reinhardtii*	–	Cu	Alleviate oxidative damage	SOD, CAT, APX	*SOD, CAT, APX*	Zheng et al., 2011
*Arabidopsis*	–	Fe	Maintain iron-homeostasis	–	*HO-1*	Kong et al., 2010

### Drought Stress

Drought stress is a widely present environmental factor which affects negatively on seed germination, seedling growth and even plant productivity. Application of exogenous hematin brought about marked increase in the activities of amylase and antioxidant enzyme such as CAT, APX, SOD, and dehydroascorbate reductase (DHAR), which were responsible for the mitigation of drought stress-induced wheat seed germination inhibition and lipid peroxidation (Liu et al., 2010; **Table 2**). To date, the investigations of CO in plant tolerance to drought stress are scarce.

### Ultraviolet Radiation Stress

The stratospheric ozone layer is thinning resulting in more ultraviolet-B (UV-B) radiation reaching the surface of the earth. UV-B exposure increases the amount of ROS and oxygen-derived free radicals, thus leading to cellular damage and apoptosis. UV-B radiation provoked an increase of the expression of HO-1 and its transcript levels in a dose-dependent manner, which was regarded as a cell protection mechanism against UV-B radiation-induced oxidative damage (Yannarelli et al., 2006). Moreover, CO production was closely related with HO-mediated heme catabolism, implying that CO probably exerted potential functions in modulating the defense response of plants to UV-B stress (Yannarelli et al., 2006; **Table 2**).

### Heavy Metals Stress

Heavy metals such as mercury (Hg), cadmium (Cd), iron (Fe), and copper (Cu) result in serious environmental pollution in many places worldwide and lead to a threat to human health and plant development. Heavy metal-induced oxidative stress in plants could be attenuated in the presence of small reactive gaseous molecules such as NO. Like NO, the vital role of CO in relieving heavy metals stress in plants has been verified (Han et al., 2007; Zheng et al., 2011). Hematin and CO supplementation to HgCl_2_-treated alfalfa root reduced lipid peroxidation and increased root elongation via activating antioxidant enzymes including glutathione reductase (GR), monodehydroascorbate reductase (MDHRR) and SOD activities, as well as decreasing lipoxygenase activity (LOX; Han et al., 2007; **Table 2**). CO also enhanced the tolerance of algae to Hg exposure which was closely related to the lower accumulation of Hg and free radical species (Wei et al., 2011; **Table 2**). The detrimental effect by Hg stress could be partially reversed by administration of CO in Indian mustard through suppressing the production of O_2_^-^ and H_2_O_2_ and increasing the accumulation of proline (Meng et al., 2011; **Table 2**). Also, Cd-induced oxidative damage was alleviated by CO pretreatment via modulating glutathione metabolism in alfalfa, which accelerated the exchange of oxidized glutathione (GSSG) to glutathione (GSH) to restore GSH: GSSG ration and further decreased the oxidative damage (Han et al., 2008; **Table 2**). In addition, Cu-induced oxidative damage in algae was alleviated by CO mainly via the improvement of CAT activity (Zheng et al., 2011; **Table 2**). Moreover, the up-regulating expression of genes related to Fe acquisition such as *AtlRT1*, *AtFRO2*, *AtF1T1*, and *AtFER1* by CO were responsible for preventing the Fe deficient-induced chlorosis and improving chlorophyll accumulation (Kong et al., 2010; **Table 2**).

## Cross-Talk between CO and other Signaling Molecules

Carbon monoxide has been highly appreciated for its versatile properties as a signaling molecule regulating diverse physiological processes in animals and plants. A number of studies have shown that CO signal transduction is extremely complex which usually doesn’t operate as the linear pathways but that extensive cross-talk occurs between various signal transduction. Thus, we provide here a brief overview of the interaction between CO signaling molecule and other signaling molecules.

### Cross-Talk between CO and NO

**FIGURE 2:**
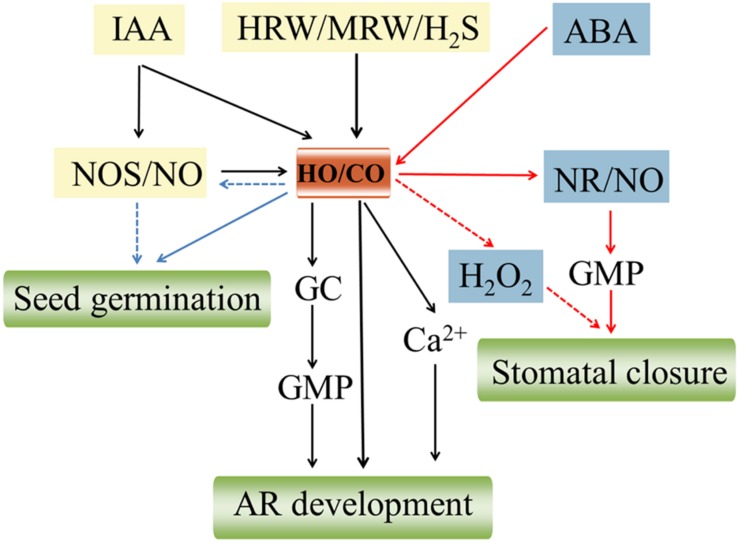
**Schematic representation of the signaling pathways involving CO and other signaling molecules in plant growth and development.** Auxin (IAA; Xuan et al., 2008), hydrogen sulfide (H_2_S; Lin et al., 2012), hydrogen-rich water (HRW; Lin et al., 2014), and MRW (Cui et al., 2015) induce the up-regulation of heme oxygenase-1/carbon monoxide (HO-1/CO), thus ultimately resulting in the adventitious root (AR) formation through activating a series of upstream signaling molecules including NO synthase/nitric oxide (NOS/NO), guanylate cyclase (GC), cyclic GMP (cGMP), and Ca^2+^. HO/CO signaling system is required for wheat seed germination probably by activating NOS/NO (Liu et al., 2010). Abscisic acid (ABA) induces CO synthetic via upregulating HO activity which triggers an overproduction of NO by (nitrate reductase) NR. Subsequently, this NO production further activates a cGMP-dependent transduction pathway, thus leading to stomatal closure (Cao et al., 2007a). Meanwhile, hydrogen peroxide (H_2_O_2_) signaling might be involved in CO induced-stomatal closure (She and Song, 2008). The red, black, and blue lines represent the signaling pathways of seed germination, AR development, and stomatal closure, respectively. The pathways using dashed lines still not fully clear.

Carbon monoxide signal transduction pathways don’t always work independently, but it is rather closely linked to NO. The two endogenously produced gasses share many common downstream signaling pathways and have some similar properties. For example, CO in animals, like NO, binded to the iron atom of heme proteins of soluble guanylate cyclase (sCG) to activate the enzyme and increase intracellular second messenger cyclic guanosine monophosphate (cGMP) production, thus exerting many of their biological functions including regulating vascular tone, inhibiting platelet aggregation, and decreasing blood pressure (Snyder et al., 1998). However, whether the phenomenon exists in plants still has no sufficient evidence to confirm. CO was also able to mimic to some extent the effect of NO in dose-dependently inducing stomatal closure (Song et al., 2008) and K-to-Na ration (Xie et al., 2008). Increasing evidence in animals supports that there exist an intimate connection in the expression of *HO* and *NOS* responsible for generating CO and NO, indicating possible interaction between the CO- and NO-generating systems. Also, it is becoming increasingly clear that CO can potentiate the activity of NO synthase (NOS) in plants. Song et al. (2008) implied that there might be existing HO-1 enzyme and NOS-like enzyme activity in *V. faba* guard cells. CO was involved in darkness-induced NO synthesis via the NOS-like enzyme (Song et al., 2008; **Figure 2**). NaCl-treated wheat seedling roots resulted in a moderate enhancement of endogenous NO level, whereas a very strong increase of NO appeared when adding 50% CO-saturated aqueous solution (Xie et al., 2008; **Figure 2**). Conversely, CO could directly bind to and inactivate NOS, decreasing the enzyme activity probably due to the competition of CO with NO for binding to its targets such as sCG (Ding et al., 1999). Interesting, recent research showed that NOS enzymes only exist in a few algal species but appear to not be conserved in land plants, suggesting the production of NO may rely mainly on nitrate assimilation in land plants (Jeandroz et al., 2016). Thus, NO synthesis is a complex process in plants and the interaction between the CO- and NO-generating systems via NOS enzymes also needs further validation. Furthermore, a functional interaction of NO and CO has been demonstrated in regulating plant growth and development. For example, CO alleviated osmotic-induced wheat seed germination inhibition and lipid peroxidation which required participation of NO (Liu et al., 2010; **Figure 2**). Santa-Cruz et al. (2010) proposed that NO was implicated in the HO signaling pathway which might directly potentiate UV-B-induced HO-1 transcription in soybean plants. Meanwhile, NO might act as a downstream signal molecule in hemin-induced cucumber AR process (Xuan et al., 2012; **Figure 2**).

### Cross-Talk between CO and Phytohormone

It has been demonstrated that CO may partially involve in IAA-induced tomato LR development via altering biosynthesis/perception in some way (Guo et al., 2008; **Figure 2**). Meanwhile, Xuan et al. (2008) strongly confirmed that there exists a serial linkage IAA→HO/CO→AR. IAA could activate HO/CO signaling system, and then triggered the signal transduction events, thus leading to AR formation in cucumber (**Figure 2**). Also, CO might be involved in ABA-induced stomatal closure which NO and cGMP may function as downstream intermediates in CO signal transduction network (Cao et al., 2007a; **Figure 2**). In addition, HO/CO might be a component or signaling system of hydrogen sulfide (H_2_S)-induced cytoprotective role against GA-induced PCD (Xie et al., 2014; **Figure 2**). Thus, phytohormone could induce various distinct developmental responses in plants, which are dependent on CO.

### Cross-talk between CO and Other Small Signaling Molecules

Similar to NO, H_2_O_2_, and other small signaling molecules also play an indispensable role in CO-mediated physiological responses. She and Song (2008) directly illustrated for the first time that CO-induced stomatal closure probably was mediated by H_2_O_2_ signaling pathways in *V. faba* (**Figure 2**). Up-regulation of *HO* expression protected aleurone layers against GA-induced PCD in wheat implicating an alteration of H_2_O_2_ metabolism (Wu et al., 2010; **Figure 2**). There is adequate evidence to support that HO/CO signaling system mediating cucumber AR formation interacts closely with H_2_S, H_2_, and CH_4_. For example, HO-1 as a downstream component was involved in H_2_S-induced AR cucumber formation through the modulation of expression of *DNAJ-1* and *CDPK1/5* genes (Lin et al., 2012; **Figure 2**). Likewise, HRW-induced AR formation was heme oxygenase-1/ CO (HO-1/CO)-dependent by up-regulating target genes related to auxin signaling and AR formation including *CsDNAJ-1*, *CsCDPK1/5*, *CsCDC6*, and *CsAUX22B/D* (Lin et al., 2014; **Figure 2**). More recently, it was suggested that MRW might serve as a stimulator of AR, which was partially mediated by HO-1/CO and Ca^2+^ pathways (Cui et al., 2015; **Figure 2**).

## Conclusion

Carbon monoxide as a gaseous signaling molecule is well studied in animals, but the current situation of CO research in plants is at an early stage. Despite the presence of CO biosynthesis in plants was first reported by Wilks (1959) and HO was claimed as its main productive route, the experimental evidence of non-enzymatic biosynthetic processes of CO is still quite limited. CO has been recognized as a signal or bio-effector involved in plant growth and development under normal and stress conditions. CO can enhance plant abiotic stress resistance in relation to the cross-talk with other signaling molecules, but the exact biological roles of CO in plants and its detail signal transduction pathway are largely unknown. Thus, more work need to be done to further elucidate the above questions by using pharmacological, physiological, and molecular approaches in the future.

## Author Contributions

All authors listed, have made substantial, direct and intellectual contribution to the work, and approved it for publication.

## Conflict of Interest Statement

The authors declare that the research was conducted in the absence of any commercial or financial relationships that could be construed as a potential conflict of interest.
